# Inactivation of Latent HIV-1 Proviral DNA Using Clustered Regularly Interspaced Short Palindromic Repeats/Cas9 Treatment and the Assessment of Off-Target Effects

**DOI:** 10.3389/fmicb.2021.629153

**Published:** 2021-05-26

**Authors:** Yufan Xu, Xiaorong Peng, Yanghao Zheng, Changzhong Jin, Xiangyun Lu, Dating Han, Haijing Fu, Chaoyu Chen, Nanping Wu

**Affiliations:** State Key Laboratory for Diagnosis and Treatment of Infectious Diseases, National Clinical Research Center for Infectious Diseases, Collaborative Innovation Center for Diagnosis and Treatment of Infectious Diseases, The First Affiliated Hospital, School of Medicine, Zhejiang University, Hangzhou, China

**Keywords:** CRISPR/Cas9, HIV-1, dual-sgRNAs, off-target effect, genome editing

## Abstract

Viral DNA integrated in host cells is a major barrier to completely curing HIV-1. However, genome editing using the recently developed technique of clustered regularly interspaced short palindromic repeats (CRISPR)/Cas9 has the potential to eradicate HIV-1. The present study aimed to use a lentiviral vector-based CRISPR/Cas9 system combined with dual-small/single guide RNAs (sgRNAs) to attack HIV-1 DNA in the latency reactivation model J-Lat 10.6 cell line and to assess off-target effects using whole-genome sequencing (WGS). We designed 12 sgRNAs targeting HIV-1 DNA, and selected high-efficiency sgRNAs for further pairwise combinations after a preliminary evaluation of the editing efficiency. Three combinations of dual-sgRNAs/Cas9 with high editing efficiency were screened successfully from multiple combinations. Among these combinations, the incidences of insertions and deletions in the sgRNA-targeted regions reached 76% and above, and no credible off-target sites were detected using WGS. The results provided comprehensive basic experimental evidence and methodological recommendations for future personalized HIV-1 treatment using CRISPR/Cas9 genome editing technology.

## Introduction

By the end of 2019, worldwide, 38 million people were estimated to be living with HIV. According to a WHO report, there were still about 1.7 million new HIV infections in 2019, and the HIV epidemic poses a major threat to global human health. A variety of drugs, such as reverse transcriptase inhibitors, protease inhibitors, and integrase strand transfer inhibitors, which have been widely used clinically, can play an antiviral role at different stages of the HIV-1 life cycle. The majority of patients with HIV/AIDS treated with these antiviral drugs were able to achieve the virological inhibition criterion that the viral load in plasma was below the detection limit (Laskey and Siliciano, 2014). However, current antiviral therapy cannot completely remove the virus from the human body because of the stable integration of proviral DNA into the cellular genome. Genome editing technology might be one of the solutions to remove proviral DNA from HIV-1 infected cells.

The clustered regularly interspaced short palindromic repeats (CRISPR)/Cas9 system, derived from the immune system of prokaryotes, which could resist the invasion of exogenous genetic material, such as phages and viruses, has become the third generation of genome editing technology (Jinek et al., 2012; Cong et al., 2013). One of the most widely used applications of the CRISPR/Cas9 system is to knockout a target gene. Its principle is that small/single guide RNA (sgRNA) targeted to the gene of interest guides Cas9 protein-mediated cleavage of an exon in the gene to break the double strand, which prompts the cells to commence non-homologous end-joining (NHEJ) repair (Mali et al., 2013). In this process, several bases are randomly added or deleted between the breakpoints. If a frame shift mutation occurs, the target gene would be inactivated.

Multiple strategies, which could be divided into two categories according to whether they act on the HIV DNA or host factors, have been proposed for HIV/AIDS treatment using the CRISPR/Cas9 system. The first category included the design of sgRNAs based on the viral genome that prompt the sgRNA-Cas9 system to attack HIV-1 DNA (Panfil et al., 2018; Das et al., 2019), and also included the strategy of “shock and kill,” which targets the HIV long-terminal repeat sequence to activate latent HIV *via* the CRISPR/dCas9 activator complex (Ji et al., 2016; Limsirichai et al., 2016; Saayman et al., 2016). The latter mainly targets the HIV co-receptor genes such as C-X-C motif chemokine receptor 4 (CXCR4) and C-C motif chemokine receptor 5 (CCR5; Liu et al., 2017, 2018; Xu et al., 2017; Yu et al., 2018). However, previous studies have voiced concerns regarding targeting to activate endogenous viral restriction factors to inhibit viral replication using CRISPR/dCas9-synergistic activation mediators, such as apolipoprotein B mRNA editing enzyme, catalytic polypeptide-like (APOBEC)3G/3B (Bogerd et al., 2015), and bone marrow stromal cell antigen 2 (BST-2; Zhang et al., 2019).

Although HIV-1 replication is inhibited by CRISPR/Cas9-induced mutations, viral escape could occur in sgRNA protected cells (Wang et al., 2016a). Another study constructed SpuT1 T cells stably expressing the Cas9 protein and dual sgRNAs, and observed the protective effect from HIV-1 LAI virus infection. The authors found that dual-sgRNAs CRISPR/Cas9 treatment could delay viral escape, block virus replication more efficiently, and even had the potential to extinguish all proviral DNAs in infected T cell cultures (Wang et al., 2016b). However, the efficiency and off-target effects of dual-sgRNAs combinations require further confirmatory studies.

Our study focused on attacking HIV-1 DNA using CRISPR therapy, for which we selected the latency reactivation model J-Lat 10.6 cell line, which contains a full-length HIV-1 genome in which the Nef gene is replaced by the green fluorescent protein (GFP) gene and has a frame-shifted, non-functional Env gene. J-Lat 10.6 cells are suitable for the *in vitro* assessment of CRISPR/Cas9-mediated targeted mutagenesis of virus genes that encode vital proteins. Using the J-Lat 10.6 HIV-1 genomic DNA as a model, we designed and verified the mutation efficiency of several dual-sgRNA combinations. In addition, to study any off-target effects of the CRISPR gene-editing system, whole-genome sequencing (WGS) was performed.

## Materials and Methods

### Cells

Human embryonic kidney (HEK) 293 T cells and J-Lat 10.6 cells were grown in Dulbecco’s modified Eagle’s medium (DMEM, Gibco, Grand Island, NY, United States) and Roswell Park Memorial Institute (RPMI)-1640 medium (Gibco), respectively. Both media contained an additional 10% fetal bovine serum (Gibco), penicillin-streptomycin (Gibco), and non-essential amino acids (Gibco). The cells were cultured at 37°C in a humidified atmosphere of 5% CO_2_.

### Plasmids

The lentiviral vector lentiGuide-Puro (plasmid #52963, Addgene, Watertown, MA, United States) and lentiCas9-Blast (Addgene; plasmid #52962) were gifts from Feng Zhang. Control sgRNA encoding oligonucleotides and those encoding sgRNAs targeting HIV-1 were ligated into the LentiGuide-Puro vector *via* its BsmB1 site, as previously described (Sanjana et al., 2014).

### Lentiviral Vector Production and Transduction

The lentiviral vector was produced and titrated as previously described (ter Brake et al., 2006). Briefly, HEK 293 T cells were transfected with a packaging plasmid (psPAX2) and the lentiviral vector plasmid (pMD2.G) using Lipofectamine 2000 (Invitrogen, Waltham, MA, United States). At 6 h after transfection, the medium was replaced and the cell culture was continued for 2 days. Then, 0.45-μm filter membranes (Millipore, Billerica, MA, United States) were used to filter the lentivirus supernatants, which were divided into aliquots and placed at −80°C. J-Lat 10.6 cells (3 × 105 cells) in culture medium were transduced with LentiCas9-Blast virus particles (30 ng of viral capsid protein p24) and cultured in the presence of blasticidin (Thermo Fisher, Waltham, MA, United States) at 4 ng/ml for 10 days to select J-Lat-cas9 cells. Subsequently, these cells (3 × 105 cells) were transduced with one or two LentiGuide-Puro virus particles (30 ng of viral capsid protein p24) and cultured in the presence of puromycin (Thermo Fisher) at 0.5 ng/ml for 10 days to produce J-Lat 10.6 cells expressing Cas9 and the sgRNAs. The dosage of two LentiGuide-Puro virus particles was prepared according to a 1:1 ratio of viral capsid protein p24 concentration.

### Genome Editing Analysis

The cellular genomic DNA was extracted using a MiniBEST Universal Genomic DNA Extraction Kit (Takara, Shiga, Japan) according to the manufacturer’s instructions. PrimeSTAR® Max DNA Polymerase (Takara) and HIV-1 specific primers (Supplementary Table S1) were used to amplify the genomic DNA using PCR. The PCR products were purified using a MiniBEST Agarose Gel DNA Extraction Kit (Takara) and were detected for T7 Endonuclease I (T7EI)-based mutations using an EnGen® Mutation Detection Kit (NEB, Ipswich, MA, United States) following the manufacturer’s protocol. The purified PCR products were cloned into a TA cloning vector and sequenced for subsequent analysis.

### Activation of the Expression of Latent HIV-1

J-Lat 10.6 cells expressing Cas9 and sgRNAs were treated with 0.1 μM of phorbol 12-myristate 13-acetate (PMA, Sigma, St. Louis, MO, United States). After 16 h of treatment, the cells were harvested and suspended in phosphate-buffered saline (PBS, HyClone, Logan, UT, United States), and GFP expression was measured using flow cytometry.

### Whole-Genome Sequencing for Off-Target Assays

Whole-genome sequencing was performed using the cellular genomic DNA to assess the potential off-target effects. GENEWIZ (South Plainfield, NJ, United States) performed the WGS and analyzed the data.

## Results

### Design of the sgRNA and the T7EI Assay

**Figure 1:**
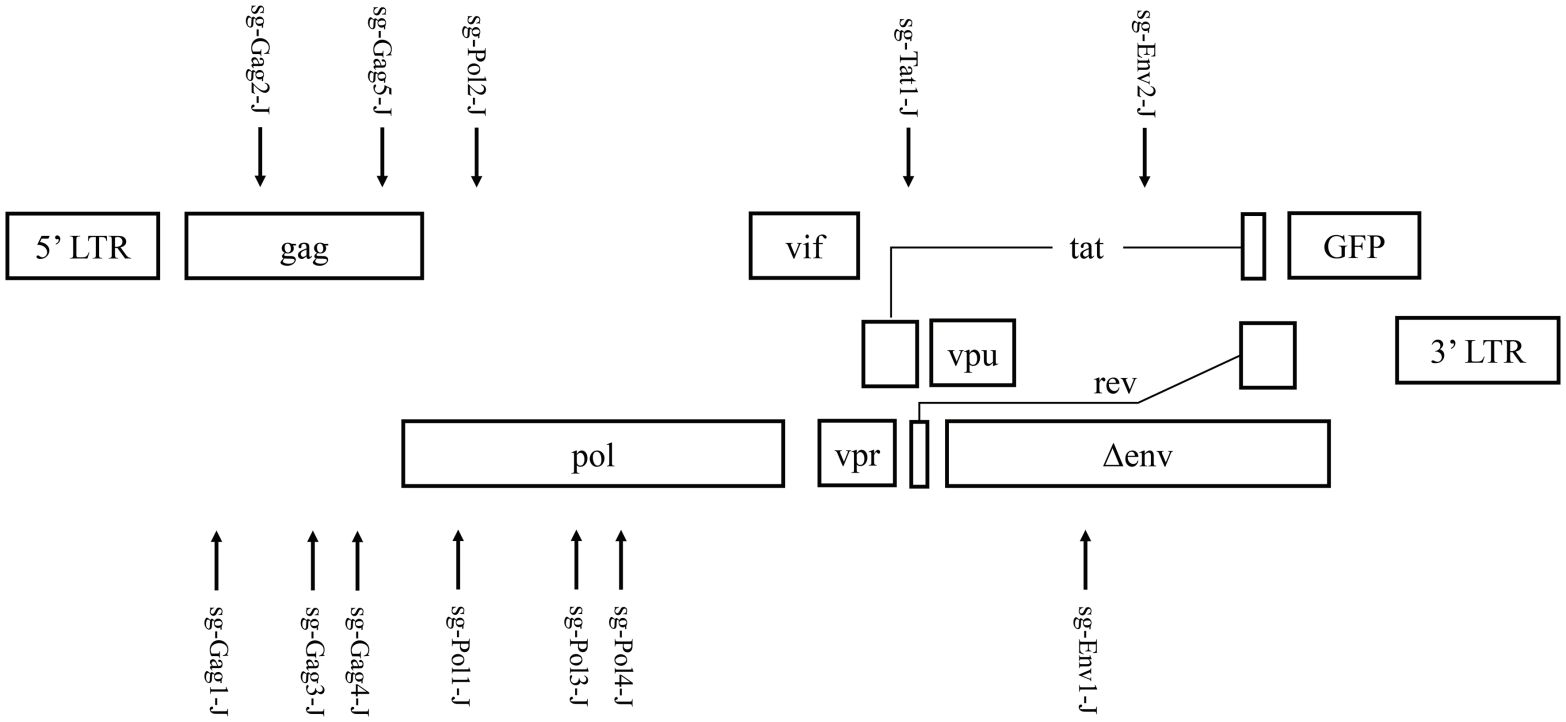
Targeting the HIV-1 genome using Cas9/single guide RNA (sgRNA) in J-Lat 10.6 cells.

**Figure 2:**
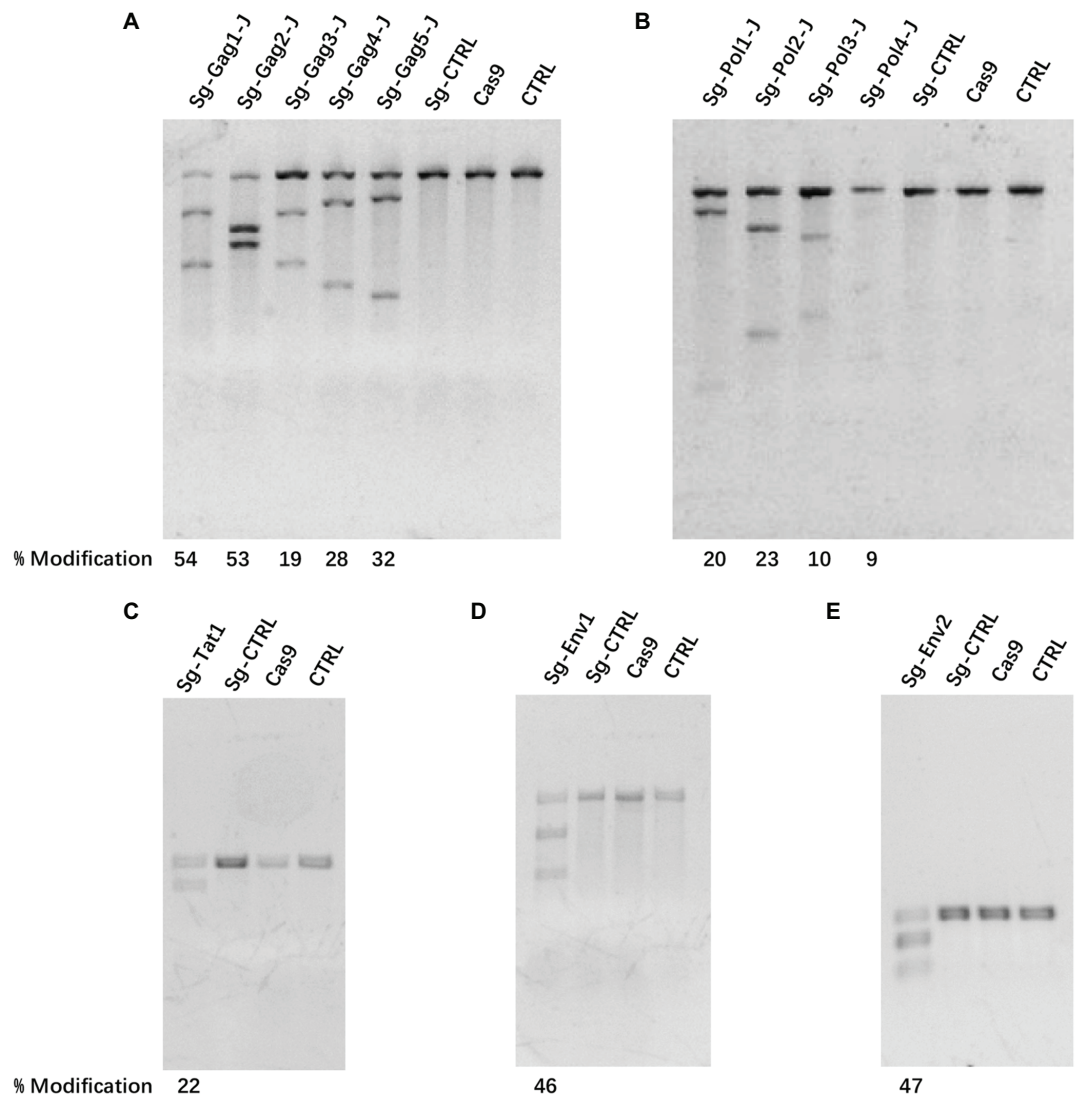
The efficiency of clustered regularly interspaced short palindromic repeats (CRISPR)/Cas9 treatment-induced cleavage of HIV-1 DNA. **(A–E)** Mutational outcomes of 12 sgRNA/Cas9 treatments were determined using a T7 endonuclease I (T7EI) assay. Cas9, cells only expressing the Cas9 protein; CTRL, cells before transfection.

Based on the latent HIV-1 genome sequence of the J-Lat 10.6 cell line, a total of 12 sgRNAs with a high on-target score, which targeted different protein-coding sequences, such as gag, pol, tat, and env genes, respectively, were designed using the online CRISPR design tool (Hsu et al., 2013; Figure 1; Table 1). Of these 12 target sites, five sgRNAs were located in the gag gene, four sgRNAs were in the pol gene, one sgRNA was in the tat gene, and two were in the env gene. Then, 12 cell lines stably expressing Cas9 and one sgRNA were constructed using lentiviral vectors. The mutation frequency of NHEJ caused by the CRISPR/Cas9 nuclease cleavage was first detected using the T7E1 assay to select effective sgRNAs. The annealed PCR product could be cleaved with T7EI. T7EI, a structure-specific enzyme, effectively identifies gene mismatches greater than 1 base. When a mismatch occurs, the two strands of the DNA molecule are cut into smaller fragments. Analyzing fragment maps could evaluate the efficiency of gene editing. The estimated mutation frequency ranged from 9 to 54% (Figure 2).

**Table 1:** Selected sgRNAs targeting HIV-1 DNA.

Name	Position/strand^a^	Guide sequence + PAM^b^	Specificity score^c^	Genomic region
sg-Gag1-J	1,185-1,207/R	CCTGGATGTTCTGCACTATA**GGG**	89	Gag (p24)
sg-Gag2-J	1,345-1,367/F	ACCATGCTAAACACAGTGGG**GGG**	74	Gag (p24)
sg-Gag3-J	1,664-1,686/R	CCGGTCTACATAGTCTCTAA**AGG**	88	Gag (p24)
sg-Gag4-J	1804-1826/R	ATTTCTTCTAGTGTAGCCGC**TGG**	92	Gag (p24)
sg-Gag5-J	1857-1879/F	CGGCCATAAGGCAAGAGTTT**TGG**	71	Gag (p24)
sg-Pol1-J	2,277-2,299/R	CCTATCTTTATTGTGACGAG**GGG**	93	Pol (Protease)
sg-Pol2-J	2,448-2,470/F	ATCTGTGGACATAAAGCTAT**AGG**	72	Pol (Protease)
sg-Pol3-J	3,018-3,040/R	ATGCTACTTTGGAATATTGC**TGG**	72	Pol (RT)
sg-Pol4-J	3,255-3,277/R	GGCTGTACTGTCCATTTATC**AGG**	88	Pol (RT)
sg-Tat1-J	5,967-5,989/R	TCTCCGCTTCTTCCTGCCAT**AGG**	63	Tat/Rev
sg-Env1-J	6,981-7,003/R	AGCAGTTGAGTTGATACTAC**TGG**	87	Env (gp120)
sg-Env2-J	7,794-7,816/F	GGAGCAGCAGGAAGCACTAT**GGG**	74	Env (gp41)
sg-CTRL	-	GGAGACGGGATACCGTCTCT**---**		-

a The position corresponds to the reference sequence HXB2. F, forward; R, reverse.

b The PAM sequence is in bold.

c The specificity score was calculated using the CRISPR design web tool from crispor.tefor.net.

### Detecting the Efficiency of Combined sgRNAs Using TA Cloning

**Figure 3:**
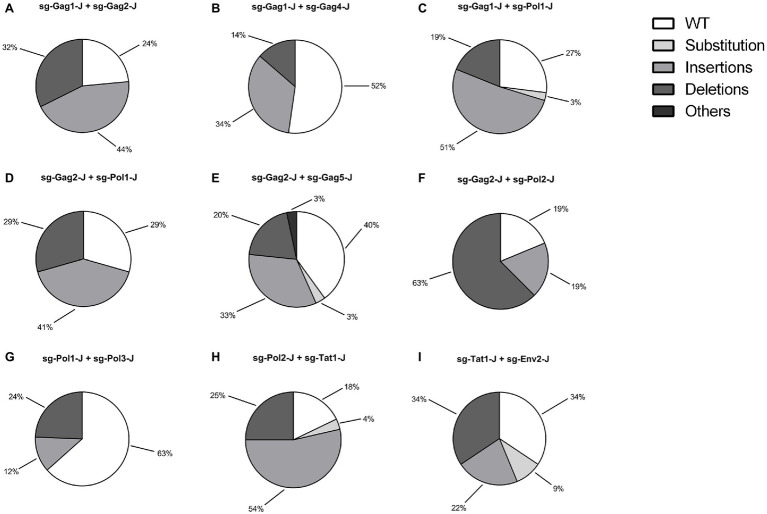
Genotypic Analysis of dual-sgRNAs/Cas9 Targeted HIV-1 DNA. (A–I) The types and frequency of mutations dual-sgRNAs/Cas9-treated in J-Lat 10.6 cells are shown. sgRNA target regions with simultaneous nucleotide substitutions (NSs) and an insertion/deletion were counted as an insertion/deletion.

**Figure 4:**
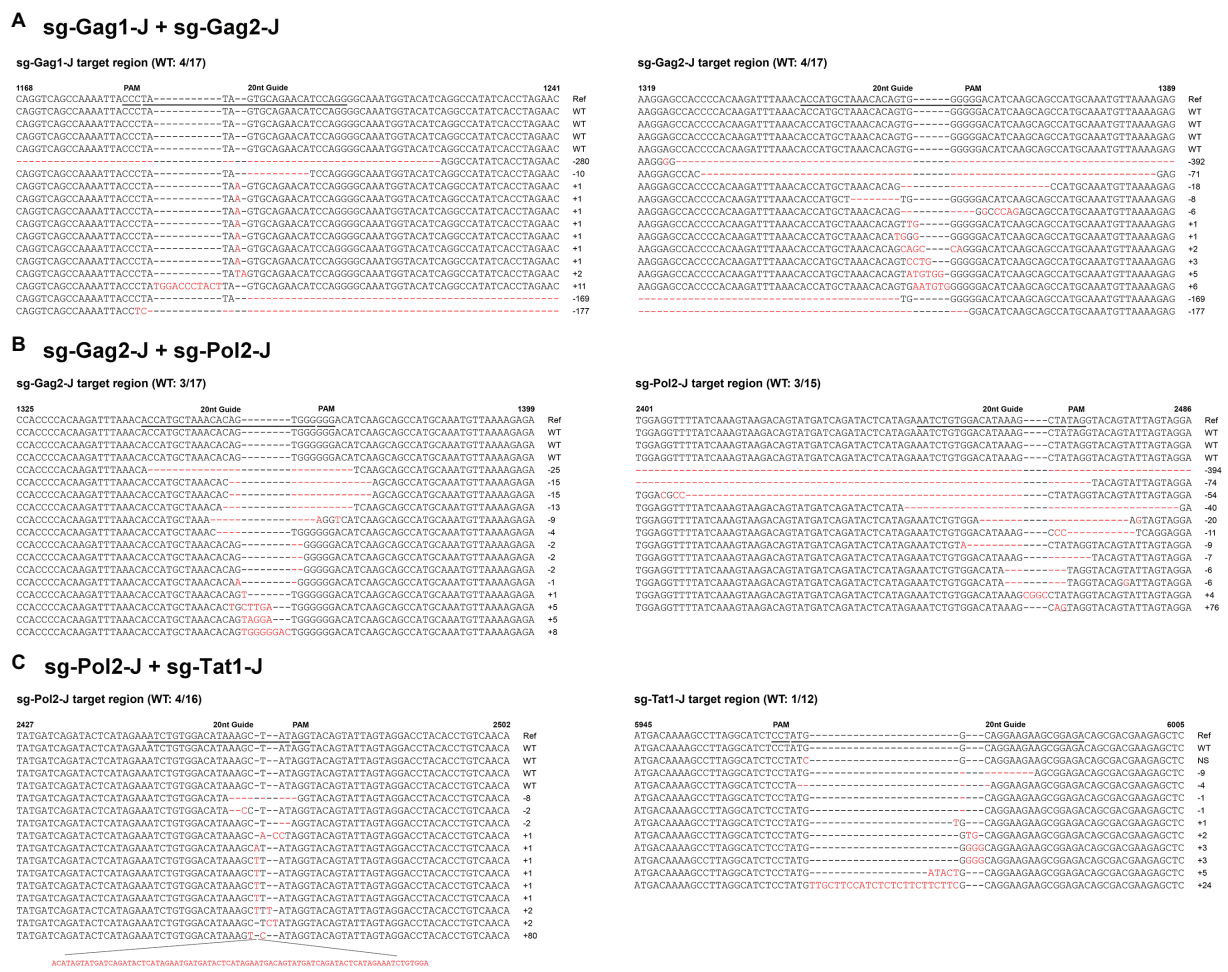
Sequence analysis of HIV-1 mutation sites in dual-sgRNAs/Cas9-treated J-Lat 10.6 cells. Features of the mutations in the three most efficient dual-sgRNA combinations: **(A)** sg-Gag1-J + sg-Gag2-J. **(B)** sg-Gag2-J + sg-Pol2-J. **(C)** sg-Pol2-J + sg-Tat1-J. All sequences were aligned with the reference strain HXB2. The 20 nt guide sequence and the protospacer adjacent motif (PAM) sequence are underlined. The sequences of nucleotide substitutions, insertions, and deletions are indicated using red letters. Ref, reference sequence; −x/+x, x nt deleted/inserted; NS, nucleotide substitution; and WT, wild-type sequence.

According to the results of the T7EI assay, sgRNAs with relatively high efficiency were selected and nine dual-sgRNA combinations, which included those targeting the same protein-coding gene and those targeting different protein-coding genes, were constructed in the lentiviral vector. The sgRNA target sites in these J-Lat 10.6 cells stably expressing Cas9 dual-guide RNAs (gRNAs) were sequenced to analyze the types and frequency of mutations. Nucleotide sequences analysis indicated that almost all of the mutations were clustered near the Cas9 cleavage site, at 3–4 nt upstream of the protospacer adjacent motif (PAM) sequence, confirming that NHEJ repair caused the mutations. The combinations with a high proportion of insertions and deletions of the HIV-1 genome were sg-Gag1-J + sg-Gag2-J (Insertions: 44%; Deletions: 32%), sg-Gag2-J + sg-Pol2-J (Insertions: 19%; Deletions: 63%), and sg-Pol2-J + sg-Tat1-J (Insertions: 54%; Deletions: 25%), respectively (Figure 3). The mutated sequences of the sgRNA-targeted regions in the three most efficient dual-sgRNA combinations are displayed in Figure 4. Mutation sequences of other combinations are shown in Supplementary Figures S1, S2. The incidence of nucleotide substitutions (NSs) was low, only being detected in four out of nine combinations, and at less than 10% (Figures 3C,E,H,I). Some interesting results were found in the sequence analysis of HIV-1 mutation sites. In the combination sg-Gag1-J + sg-Gag2-J, we found deletion of the entire viral DNA between the two sgRNA-targeted sites in two cases, with 169 and 177 bp being deleted, respectively (Supplementary Figure S3). A chromosomal inversion occurred between two sgRNA-targeted sites in the combination sg-Gag2-J + sg-Gag5-J (Supplementary Figure S4).

### Blocking of Latent HIV-1 Expression

**Figure 5:**
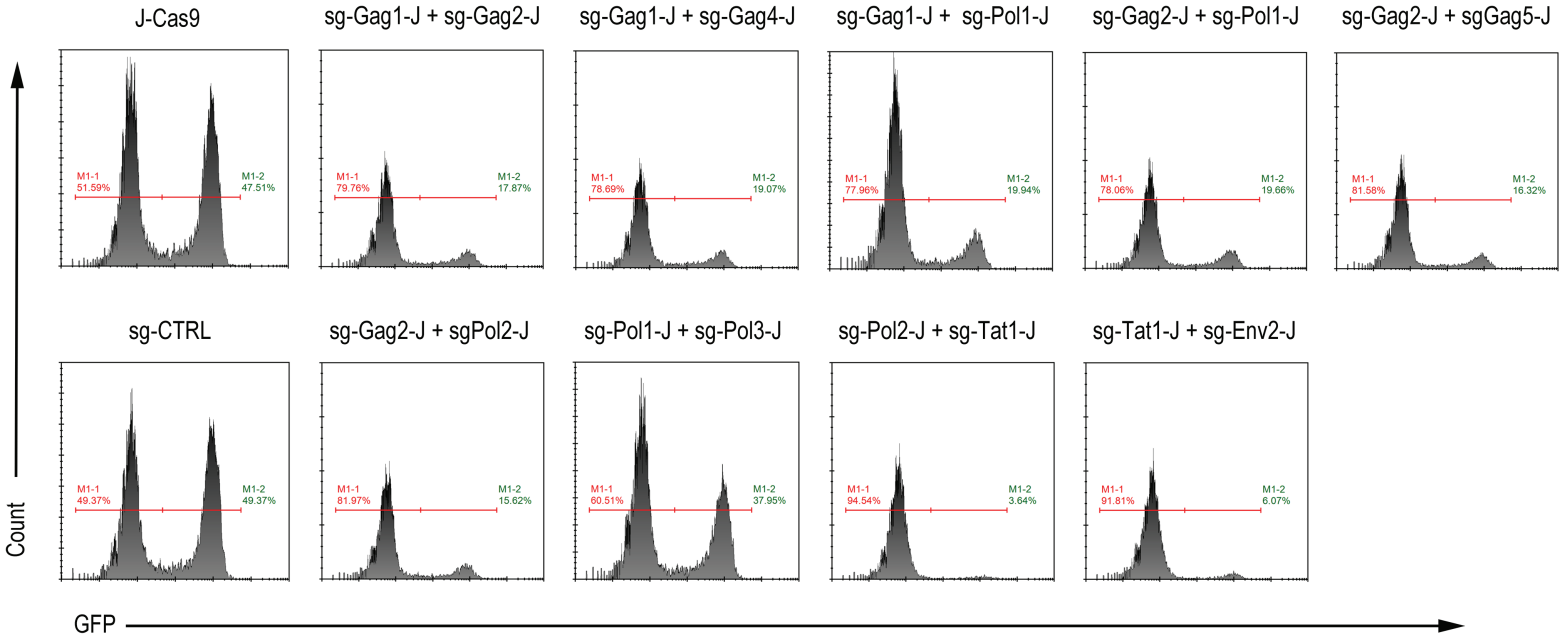
Activation of latent HIV-1 expression in J-Lat 10.6 cells expressing Cas9 and dual-sgRNAs. M1-2 represents the percentage of GFP-positive cells.

In addition, we performed a test to activate latent HIV-1 in the J-Lat 10.6 cells stably expressing Cas9 and dual-sgRNAs. After 16 h of treatment with 0.1 μM PMA, GFP expression was monitored using flow cytometry. Except for the sg-Pol1-J + sg-Pol3-J combination, for which the inhibition effect was not obvious because of the low incidence of insertions and deletions in the HIV-1 genome (Figure 3G), the GFP expression of the other eight combinations was significantly inhibited, among which the sg-Pol2-J + sg-Tat1-J combination showed the most significant inhibitory effect (Figure 5).

### Assessment of Off-Target Effects Using WGS

**Figure 6:**
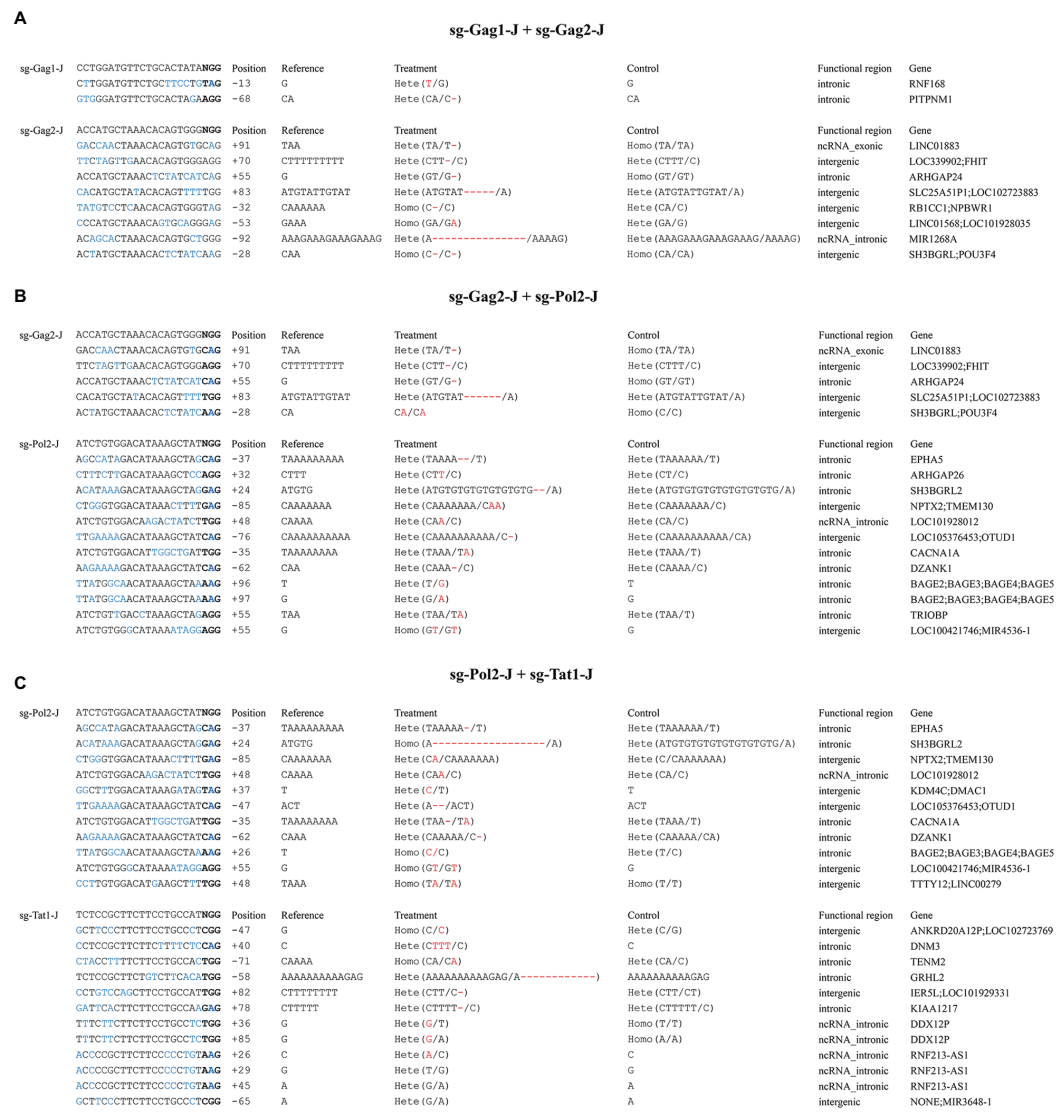
Analysis of potential off-target sites using whole-genome sequencing (WGS). **(A)** sg-Gag1-J + sg-Gag2-J. **(B)** sg-Gag2-J + sg-Pol2-J. **(C)** sg-Pol2-J + sg-Tat1-J. In the potential off-target regions, mismatched bases to the sgRNAs are shown in blue and the bases in bold represent the PAM sequence; the position of the mutation sites is indicated by the relative distance to the 1st base (+1) of the 20 nt complementary regions; the differential mutations between the control and the treatment groups are highlighted in red.

We selected three combinations with relatively high editing efficiency (sg-Gag1-J + sg-Gag2-J, sg-Gag2-J + sg-Pol2-J, and sg-Pol2-J + sg-Tat1-J), with the untreated J-Lat 10.6 cell as controls, to perform WGS (30x coverage) and detect single nucleotide variants (SNVs) and insertions and deletions (Indels). First, the SNV/Indel of a single treatment group was compared with the control to identify unique the mutation sites of the treatment group, and the upstream and downstream 100 bp sequences of these sites were obtained from the reference genome (GRCh37/hg19) as candidate potential off-target regions. Next, we aligned the sgRNA sequence with the candidate potential off-target regions, and filtered out the regions with more than six mismatched bases and those not containing the PAM (NRG) sequence. Finally, the potential off-target regions of three treatment groups after screening were identified, and all of these regions were located in intergenic or intronic regions (Figure 6). By determining the relative position between the mutation site and the 20 nt complementary region of the sgRNA in all potential off-target regions, we found no mutation sites located in the 20 nt complementary regions (the relative position between +1 and +20).

## Discussion

In this study, among the 12 sgRNA sequences designed, sg-Tat1-J and sg-Env2-J refer to previously reported sequences because of their high activity in targeting HIV-1 DNA (Wang et al., 2016b), and the viral transcriptional repressive effect was significant, especially when targeting the sg-Tat1-J site. According to the results of the T7EI assay, sgRNAs with a low mutation rate were not included in the combination treatments. We selected three combinations with a high incidence of insertions and deletions from nine dual-sgRNA combinations, and then performed WGS to detect any off-target effects. A variety of studies have confirmed the effectiveness of CRISPR/Cas9 system in eliminating HIV-1 DNA (Ebina et al., 2013; Hu et al., 2014; Liao et al., 2015; Zhu et al., 2015; Bella et al., 2018; Mefferd et al., 2018; Ophinni et al., 2018; Wang et al., 2018; Yin et al., 2018); however, few of them detected off-target effects using deep sequencing to assess the safety of the method. Targeting HIV-1 DNA was expected to have the advantage of inducing a low frequency of off-target mutations, because theoretically, an editing system targeting viral DNA would not affect the host genes. The effectiveness and safety of a treatment are equally important. Therefore, evaluation of off-target effects is essential for any gene editing therapy. Compared with detection methods involving directly amplifying the predicted off-target sites and sequencing by Sanger sequencing or next-generation sequencing, WGS is more comprehensive to assess off-target effects because it can simultaneously detect coding and non-coding regions. Several studies have used WGS to detect off-target effects edited by the CRISPR/Cas9 system (Feng et al., 2014; Ghorbal et al., 2014; Li et al., 2018; Lyu et al., 2018); however, higher sequencing depth is required and the cost is relatively high. In the present study, we used WGS to assess off-target effects on three combined samples with high efficiency. Although many mutations were detected as potential off-target regions in the experimental groups, these mutation sites were not located within the 20 nt complementary region of the sgRNA. Therefore, these mutations are not credible off-target sites.

In the last few years, studies targeting HIV-1 co-receptor CCR5 using CRISPR-Cas9 have made significant progress, such as CCR5 disruption in induced pluripotent stem cells (Kang et al., 2015), inducing CCR5 Δ32/Δ32 homozygotes in CD4+ Jurkat cells and primary CD4+ cells (Qi et al., 2018), and even CCR5-Edited stem cells in a patient with HIV-1 and acute lymphocytic leukemia (Xu et al., 2019). However, there is a limitation in editing the *CCR5* gene, in that it can only prevent CCR5-tropic HIV-1 virus infection, but not CXCR4-tropic HIV-1 virus. In addition, in terms of safety, compared with directly attacking viral DNA using CRISPR/Cas9, the short and long-term effects on normal immune function caused by inactivation of *CCR5* by CRISPR/Cas9 genome editing technology require further research.

HIV-1 can be divided into many subtypes and recombinant subtypes, and the distribution of these subtypes varies in different countries and regions (Maartens et al., 2014). Genetically diverse viral populations still exist in the same subtype and even within each infected individual (Hemelaar, 2012). Therefore, it is a challenge to design general and conserved sgRNA sequences for all subtypes of HIV-1, which are both effective and safe (Roychoudhury et al., 2018). With the development of CRISPR/Cas9 genome editing technology, targeting HIV-1 DNA could be individualized *via* sgRNA design in the future (Dampier et al., 2018). The research object in this study was a cell model of latency; therefore, we did not need to consider the genetic diversity of HIV-1. If the object of further research is primary CD4+ cells in HIV-1 infected patients, the viral DNA sequence should be amplified and sequenced using deep sequencing. Multiple individualized high score sgRNAs should be designed, which should avoid the sites with a high mutation rate as much as possible (Dampier et al., 2018). Then, the efficacy and safety of the sgRNAs combinations could be verified *in vitro*, and the most appropriate combinations for individuals would be selected for CRISPR treatment. However, how to locate all the latently infected cells present in long-lived HIV-infected individuals and how to deliver the gene editing tools to these latently-infected cells remain a challenge.

In the present study, we found deletion of entire viral DNA and a chromosomal inversion between the two sgRNAs-targeted sites. These mutations seriously disrupted the integrity of the viral genome and led to loss of viral protein function. Thus, the dual-sgRNA combinations, consisting of two efficient and safe sgRNAs located between hundreds of bases in a protein-coding gene of HIV-1, could become a desirable and popular strategy to destroy the viral genome. Nonetheless, the low incidence of fragment excision and fragment inversion was an important issue that requires further attention (Binda et al., 2020). In addition to the dual-sgRNAs CRISPR treatment strategy, according to a recent study (Dash et al., 2019), a combination therapy comprising long-acting antiviral drugs and CRISPR treatment targeting HIV-1 DNA could lead to the safe elimination of HIV-1 infection, possibly representing a future cure for HIV-1. By contrast, CRISPR treatment targeting HIV-1 DNA combined with RNA interference can lead to cross-resistance (Zhao et al., 2017). Therefore, more studies are required to confirm whether CRISPR treatment combined with other treatments is appropriate.

A novel Adeno-associated virus (AAV)-based vector (AAV9P1) with a synthetic surface peptide can deliver CRISPR/Cas9 system to astrocytes and might facilitate inactivation of persisting HIV-1 proviruses (Kunze et al., 2018), which is of great significance to eliminate viral reservoirs in the central nervous system. AAV is also a promising tool for gene delivery *in vivo*. Excision of HIV-1 proviral DNA in solid tissues and organs in humanized mice with chronic HIV-1 infection can be achieved *via* AAV-delivered CRISPR/Cas9 (Yin et al., 2017). According to a recent study, AAV9-CRISPR/Cas9 gene editing was designed to eliminate simian immunodeficiency virus (SIV) proviral DNA in ART-treated non-human primates (Mancuso et al., 2020). These findings offer a significant step toward the elimination of HIV-1 reservoirs in the clinic. Notably, CRISPR-Cas13a could inhibit HIV-1 infection by directly targeting viral RNA and diminishing viral gene expression (Yin et al., 2020). RNA editing technology has the potential to treat HIV-1 infection; however, the off-target effects cannot be ignored (Mao et al., 2019).

## Conclusion

We designed and verified multiple sgRNAs and combinations comprising dual-sgRNAs that targeted HIV-1 genomic DNA in a cell line model of latency. WGS was used to assess off-target effects in the three efficient combinations selected, and no reliable off-target sites were detected. The study provided a comprehensive experimental basis and methodological suggestions for CRISPR/Cas9 individualized treatment of HIV-1 in the future, from the design of sgRNAs to the selection of efficient combinations and verification of safety. On the premise of effectiveness and safety, the dual-sgRNAs CRISPR/Cas9 treatment targeting HIV-1 protein-coding genes could serve as a therapeutic procedure to achieve a cure and epidemic control of HIV/AIDS.

## Data Availability Statement

The original contributions presented in the study are included in the article/Supplementary Material, further inquiries can be directed to the corresponding author.

## Author Contributions

YX and XP performed the research, analyzed the data, and wrote the manuscript. NW designed the research and revised the manuscript. YZ, CJ, XL, DH, HF, and CC performed the research. All authors read and approved the final version of the manuscript.

### Conflict of Interest

The authors declare that the research was conducted in the absence of any commercial or financial relationships that could be construed as a potential conflict of interest.

## Supplementary Material

Supplementary Figure S1 Analysis of the sequences of HIV-1 mutation sites in dual-sgRNAs/Cas9-treated J-Lat 10.6 cells. Feature of the mutations in the three most efficient dual-sgRNA combinations: **(A)** sg-Gag1-J + sg-Gag4-J **(B)** sg-Gag1-J + sg-Pol1-J **(C)** sg-Gag2-J + sg-Pol1-J. All sequences were aligned with the reference strain HXB2. The 20 nt guide sequence and the PAM sequence are underlined. The sequences of nucleotide substitutions, insertions, and deletions are indicated using red letters. Ref, reference sequence; −x/+x, x nt deleted/inserted; NS, nucleotide substitution; WT, wild-type sequence.

Supplementary Figure S2 Sequence analysis of HIV-1 mutation sites in J-Lat 10.6 cells treated with dual-sgRNAs/Cas9. Features of the mutations in the three most efficient dual-sgRNA combinations: **(A)** sg-Gag2-J + sg-Gag5-J **(B)** sg-Pol1-J + sg-Pol3-J **(C)** sg-Tat1-J + sg-Env2-J. All sequences were aligned with the reference strain HXB2. The 20 nt guide sequence and the PAM sequence are underlined. The sequences of nucleotide substitutions, insertions, and deletions are indicated using red letters. Ref, reference sequence; −x/+x, x nt deleted/inserted; NS, nucleotide substitution; WT, wild-type sequence.

Supplementary Figure S3 Deletion of the entire viral DNA between two sgRNA-targeted sites in the combination sg-Gag1-J + sg-Gag2-J **(A)** 169 bp deletion **(B)** 177 bp deletion with two single-base substitutions.

Supplementary Figure S4 Chromosomal inversion between two sgRNA-targeted sites in the combination sg-Gag1-J + sg-Gag5-J.
