# Supplementary figures and images for: Inactivation of Latent HIV-1 Proviral DNA Using Clustered Regularly Interspaced Short Palindromic Repeats/Cas9 Treatment and the Assessment of Off-Target Effects
Source: Front Microbiol. 2021 May 26;12:629153. doi: 10.3389/fmicb.2021.629153 (PMC8187572; doi:10.3389/fmicb.2021.629153)

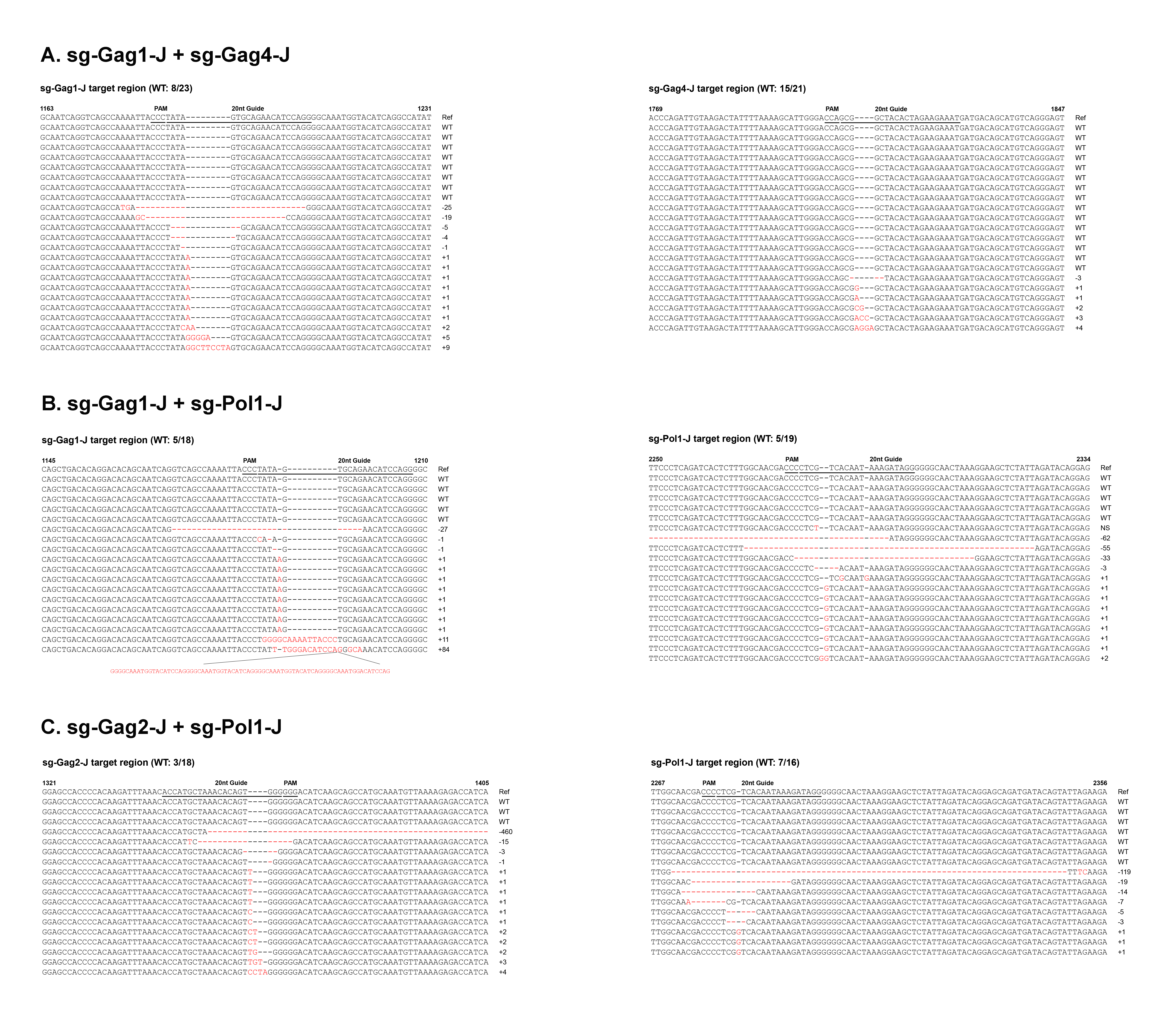

Supplement: Supplementary Figure S1 — Analysis of the sequences of HIV-1 mutation sites in dual-sgRNAs/Cas9-treated J-Lat 10.6 cells. Feature of the mutations in the three most efficient dual-sgRNA combinations: (A) sg-Gag1-J + sg-Gag4-J (B) sg-Gag1-J + sg-Pol1-J (C) sg-Gag2-J + sg-Pol1-J. All sequences were aligned with the reference strain HXB2. The 20 nt guide sequence and the PAM sequence are underlined. The sequences of nucleotide substitutions, insertions, and deletions are indicated using red letters. Ref, reference sequence; −x/+x, x nt deleted/inserted; NS, nucleotide substitution; WT, wild-type sequence. [file Image_1.TIF]

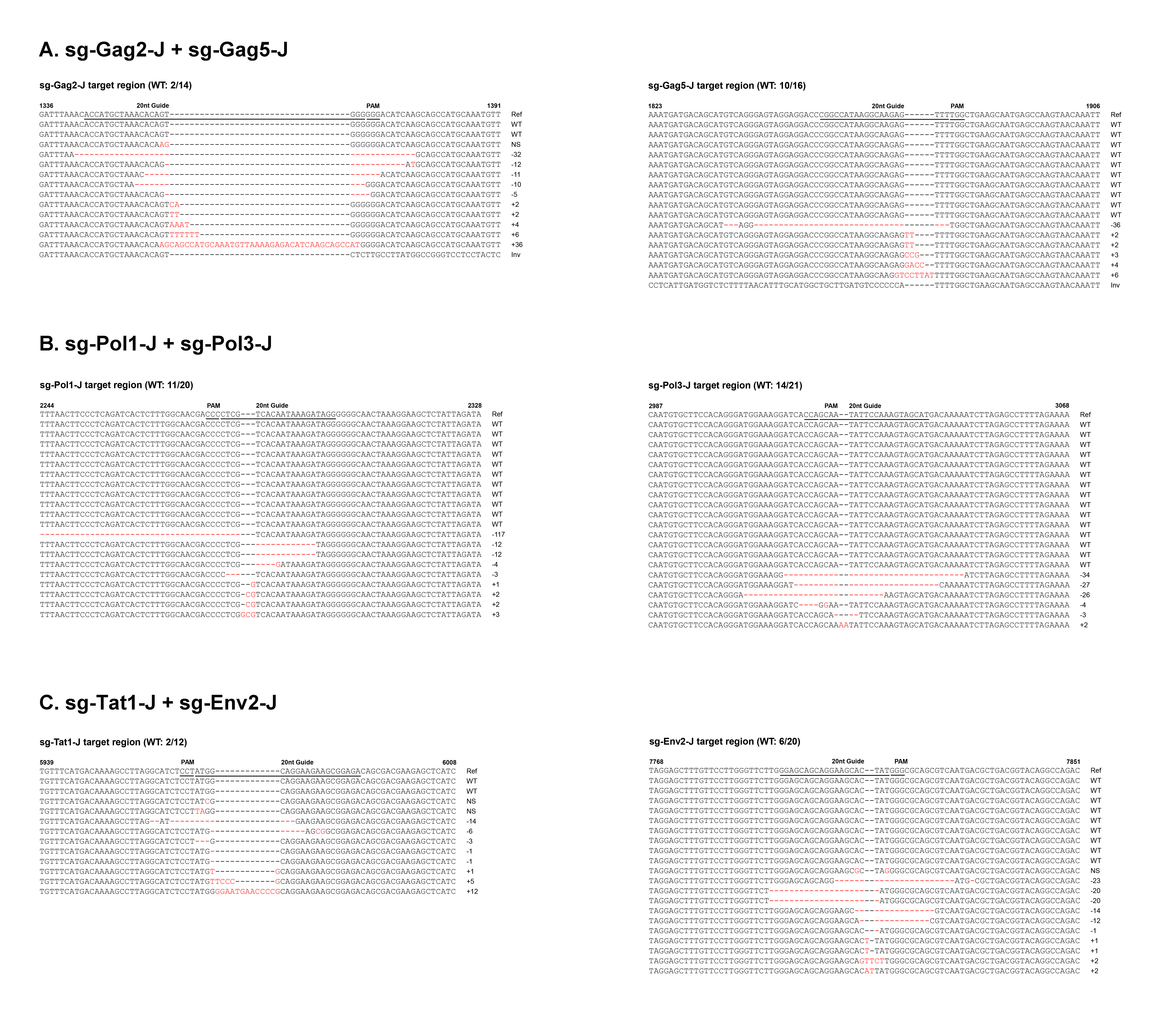

Supplement: Supplementary Figure S2 — Sequence analysis of HIV-1 mutation sites in J-Lat 10.6 cells treated with dual-sgRNAs/Cas9. Features of the mutations in the three most efficient dual-sgRNA combinations: (A) sg-Gag2-J + sg-Gag5-J (B) sg-Pol1-J + sg-Pol3-J (C) sg-Tat1-J + sg-Env2-J. All sequences were aligned with the reference strain HXB2. The 20 nt guide sequence and the PAM sequence are underlined. The sequences of nucleotide substitutions, insertions, and deletions are indicated using red letters. Ref, reference sequence; −x/+x, x nt deleted/inserted; NS, nucleotide substitution; WT, wild-type sequence. [file Image_2.TIF]

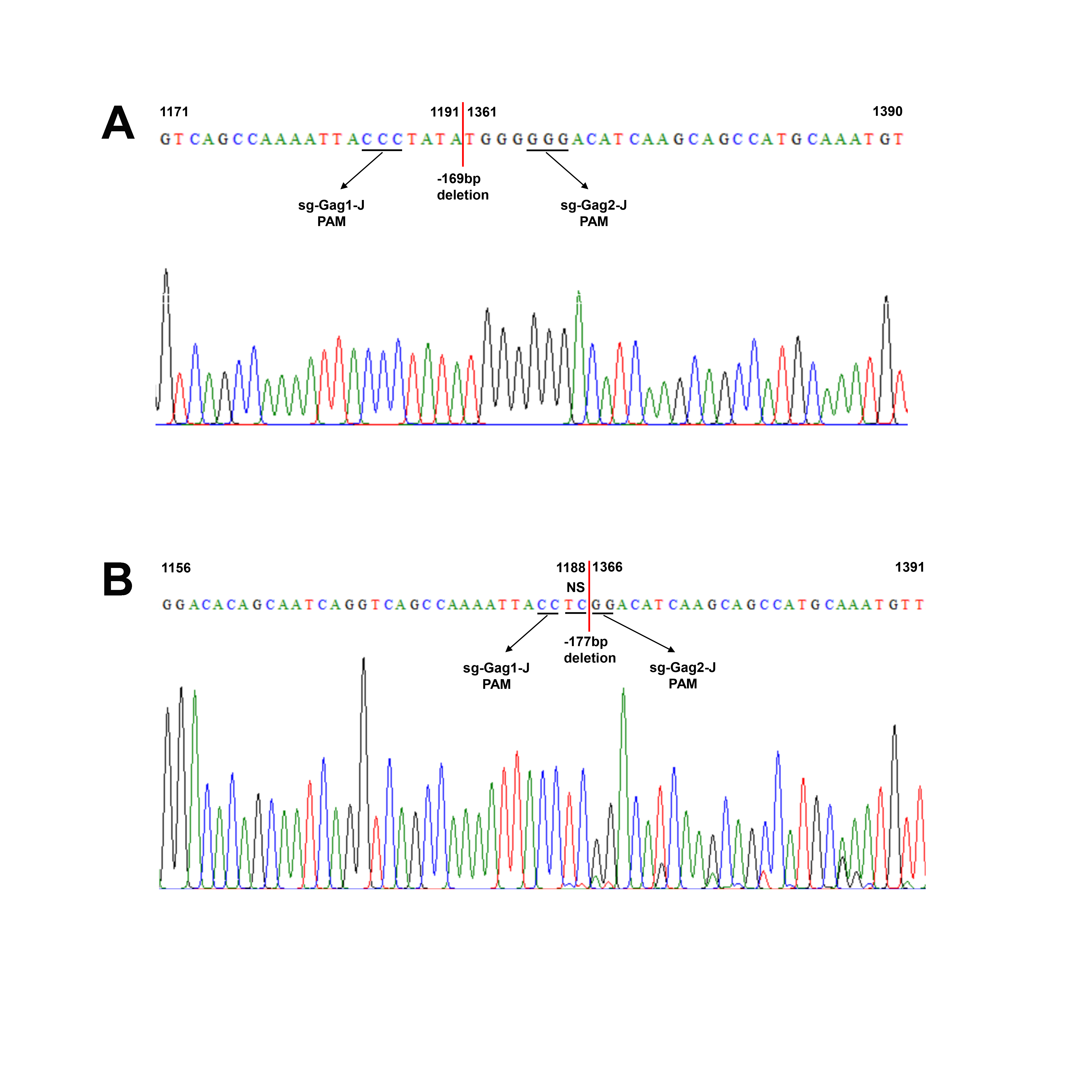

Supplement: Supplementary Figure S3 — Deletion of the entire viral DNA between two sgRNA-targeted sites in the combination sg-Gag1-J + sg-Gag2-J (A) 169 bp deletion (B) 177 bp deletion with two single-base substitutions. [file Image_3.TIF]

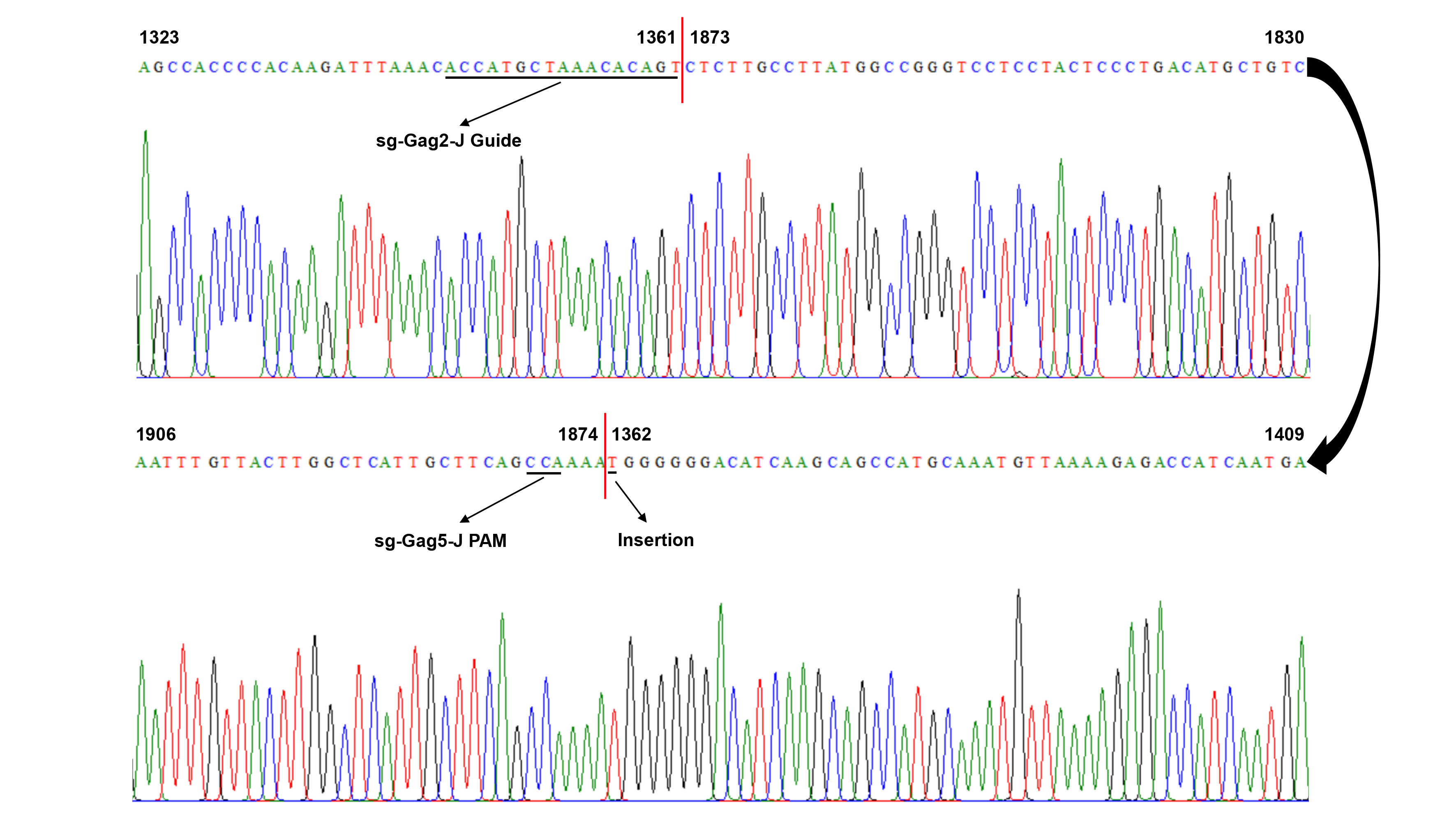

Supplement: Supplementary Figure S4 — Chromosomal inversion between two sgRNA-targeted sites in the combination sg-Gag1-J + sg-Gag5-J. [file Image_4.TIF]
