# Supplementary material for: Inactivation of Latent HIV-1 Proviral DNA Using Clustered Regularly Interspaced Short Palindromic Repeats/Cas9 Treatment and the Assessment of Off-Target Effects
Source: Front Microbiol. 2021 May 26;12:629153. doi: 10.3389/fmicb.2021.629153 (PMC8187572; doi:10.3389/fmicb.2021.629153)
Supplement: Supplementary file 5 [file Table_1.docx]

| **TABLE S1.** HIV-1-speciﬁc primers used for the amplification of sgRNA target regions. | | | |
| --- | --- | --- | --- |
| **Target region** | **Name** | **Sense primer (5′ to 3′)** | **Antisense primer (5′ to 3′)** |
| gag | sg-Gag1-J | CTTAAGCCTCAATAAAGCTTGCCTTGA | TACTGTATCATCTGCTCCTGTATC |
|  | sg-Gag2-J |  |  |
|  | sg-Gag3-J |  |  |
|  | sg-Gag4-J |  |  |
|  | sg-Gag5-J |  |  |
| pol | sg-Pol1-J | TGGAAATGTGGAAAGGAAGGAC | CTGTATTTCTGCTATTAAGTCTTTTGA |
|  | sg-Pol2-J |  |  |
|  | sg-Pol3-J |  |  |
|  | sg-Pol4-J |  |  |
| tat | sg-Tat1-J | ATATCAAGCAGGACATAACAAGG | CTATGATTACTATGGACCACACA |
| gp120 | sg-Env1-J | ATGGGATCAAAGCCTAAAGCCATGTG | CCTTGGTGGGTGCTACTCCTAATGGTTCA |
| gp41 | sg-Env2-J | GCACCCACCAAGGCAAAGAGAAGAGTGG | CAACCCCAAATCCCCAGGAGCTGTTGATCC |
